# A novel surgical technique for prevention of self-sucking in cattle and buffaloes: tongue piercing

**DOI:** 10.1186/s12917-022-03283-8

**Published:** 2022-05-20

**Authors:** Yasser Salman, Mohamed Semieka, Mohamed Karmi, Al-lethie A. Al-lethie

**Affiliations:** 1 Department of Surgery, Anesthesiology, and Radiology, Faculty of Veterinary Medicine, Aswan University, Aswan, 81528 Egypt; 2 Department of Surgery, Anesthesiology, and Radiology, Faculty of Veterinary Medicine, Assiut University, Assiut, 71526 Egypt; 3 Department of Food Hygiene, Faculty of Veterinary Medicine, Veterinary Medicine, Aswan University, Aswan, 81528 Egypt

**Keywords:** Cattle, Buffaloes, Self-sucking, Tongue piercing

## Abstract

**Background:**

Self-sucking is an abnormal behavior lead to important economic losses for dairy farms. The objective of this study was to evaluate tongue piercing as a novel technique to prevent self-sucking in cattle and buffaloes. The study was carried out on 26 cows and 4 buffaloes suffered from self-sucking. Tongue piercing was achieved by the application of an implant in the midline of the tongue and anterior to the frenulum linguae. With a follow up period of 6 months.

**Results:**

This implant produced mechanical disruption of the affected animals ability to curl their tongues, in a U- shape manner, subsequently it was impossible for these animals to cup their tongues and suck its own teats. Slight swelling around the piercing site of the tongue was observed among all animals on the first 3 days after surgery. No other complications have been reported.

**Conclusion:**

Tongue piercing is an effective, rapid, easy, minimally invasive technique to prevent self-sucking among cattle and buffaloes, moreover, the technique was more widely acceptable by the owners than other traditional and surgical methods.

## Background

Self-sucking is an abnormal behavior in dairy farms in which the affected animals curl their tongues into a U shape for the purpose of sucking milk from their own teats [1].

Various factors can influence the occurrence of self-sucking, including feeding management, nutrient deficiencies, housing systems and genetic factors [2–5].

Self-sucking causes significant financial losses for dairy farms by lowering milk yield and causing udder damage and mastitis. Moreover, the affected animal can be left out of breeding in early periods due to only this kind of behavioral defect [5].

To prevent self-sucking, a variety of conservative methods (halters or cradles, pronged nose rings, weaning rings, and nose flaps) were used, but these methods are ineffective as the relapse rate is considerable, and may cause severe injuries to the affected animal as well as neighboring animals. Additionally, the affected animal regarded as of lower quality in markets [6–8]. Because of the failure of these methods to solve the problem, their complications, and their unacceptability by the owners, surgical treatment became the most dependable and radical solution for preventing self-suckling in cattle and buffaloes [3].

A variety of surgical techniques were used for the prevention of self-sucking. These techniques were designed to prevent animals from being able to cup the dorsum of their tongues and suck teats, including the ventral glossectomy technique [7–10] and the lateral glossectomy technique [10, 11]. Recently, two less-invasive techniques were performed by applying silk stitches to the free portion of the tongue [12, 13]. However, these techniques varied in their success rates. Some complications have been reported, for instance, excessive swelling, infection, decreased feeding and milking, and even culling. This prompted us to find a better surgical technique to prevent this abnormal behavior.

The purpose of this research was to assess the tongue piercing technique as a new method for the prevention of self-sucking in cattle and buffaloes.

## Results

### Pre-operative observations

Physiological parameters in all the affected animals were sufficiently normal for performing surgery. All vital parameters remained within the normal physiological range after performing the procedure.

### Intra-operative observations

Slight bleeding was observed at the puncture site of the trocar insertion; however, no other intra operative complications were recorded. The blood vessels and nerves of the tongue were not invaded and remained intact.

### Post-operative observations

There was no further bleeding after the tourniquet was removed. Slight swelling around the tongue piercing site was observed in all animals during the first 3 days after surgery.

In all treated cases, the animals’ normal prehension of all types of feed was unaffected, and all animals were able to eat and drink properly immediately after the surgical procedure.

Operated animals attempted unsuccessful self-suckling for 1–2 weeks after surgery, but by the end of the second week, they had completely stopped.

### Long-term observations and efficacy

A six-month follow-up observation revealed that, clinically, neither local nor systemic signs of inflammation or infection could be observed. There were no problems with the device that was used. Self-sucking had completely disappeared in all of the treated animals.

## Discussion

Self-sucking is a common problem in dairy animals, resulting in milk loss as well as udder damage, mastitis, and breeding animal culling [14].

In the present study, despite several trials of isolation and application of bull rings with spikes, the problem persisted and there was a great fear of the spread of this anomalous behavior by imitation from the neighboring cows. This agrees with Abou-El-Ella [7] who stated that due to the failure of all traditional control methods such as isolation and application of bull rings with spikes, the surgical interventions are the last trial to resolve the problem and the long-term benefits from the surgical procedures are more satisfactory than the conservative methods.

The previous glossectomy techniques for treatment of this problem relied on the surgical excision of varying thicknesses of tongue tissues to disrupt the tongue’s contour and prevent it from curling, making milk suction difficult. The procedures of these techniques are time- consuming, may necessitate general anesthesia and result in tongue tissue damage, which can lead to bleeding and sepsis. Furthermore, determining the precise size and thickness of the tongue tissue to be excised is a major challenge with these techniques [12].

El-Sherif and Seddek et al. [12, 13] performed less-invasive surgical methods by applying silk stitches to the tissues of the tongue to make the tongue’s dorsal surface convex to prevent this abnormal behavior. These techniques had a low success rate in the long term because the amount of tongue tissue involved in the stitches was less than the minimum amount required to make a change in the tongue contour, so the animals could theoretically continue sucking after surgery [8–10]. Furthermore, prolonged use of non-absorbable multifilament braided suture material with a high capillary ascension in the moist environment of the oral cavity promotes bacterial infections, which could lead to glossitis or the formation of a tongue abscess [15, 16].

Body piercing is the insertion of an ornament into openings in the skin or mucosa [17]. Body piercing has been used as a type of body adornment since ancient times, both for ritual or aesthetic reasons, as well as to declare one’s membership in a particular social or ethnic group. Piercing is now very popular among young adults and teenagers as a means of self-expression [18]. Despite the fact that the ear is still the most popular piercing location, the orofacial area, which includes the nose, lips, cheeks and, in particular, the tongue, is growing in popularity [19]. There are various forms of tongue piercings and the most common forms are barbells, rings or studs of different lengths and thicknesses that are inserted through the tongue. It is most commonly performed in the midline of tongue, but it can also be performed laterally to the midline [20].

In the current study, the piercing implant created mechanical disruption of the affected animals’ ability to curl their tongues in a U-shape, rendering these animals unable to cup their tongues and suck milk from their own teats. Here, the tongue has the ability to perform its function efficiently after the operation and all animals were allowed to drink and eat freely after the operation compared with other surgical techniques. The food was offered after 12 hours in the sublingual-mucosal-resection technique and after 24 hours in the partial glossectomy technique. This may be attributed to the post-operative pain associated with these techniques, which dissuades the animals from feeding [21]. On the other hand, the old mechanical devices, such as cradles, weaning rings, and noseflap-halters, could result in severe injuries to the affected animal as well as neighbouring animals. Furthermore, the rate of behaviour relapse is high (ranging from 9 to 55%), and feeding and drinking may be reduced [9].

In the present study unsuccessful attempts of self-suckling were made by operated animals for 1–2 weeks after surgery, but by the end of the second week, they had entirely stopped. A six-month follow-up observation revealed that self-sucking had completely disappeared in all of the treated animals. In the previous studies, the animals’ trials of self-sucking lasted 3 weeks in the intra-lingual suture pattern technique [13] and more than 4 weeks were needed for complete healing in glossectomy techniques [7].

In this study, tongue piercings are performed with a sheep stainless trocar with cannula and the implant consists of a barbell with two screws; one is fixed and the other is removable. In humans, barbells are the most common implant placed in the tongue after it has been pierced with a hollow needle. It is made up of a stem that varies in length and has a ball-shaped tip on each end [22].

In humans, tongue implants are made from a variety of different materials such as surgical stainless steel, silver and gold, as well as synthetic materials such as Teflon, nylon, and plastic. Recently, implants made of natural materials such as stone, wood, horn, ivory and bone have been developed [23]. The implant utilized in this study was made of Teflon material, which is one of the most biocompatible materials used in the biomedical field; it is stable in host tissues and does not elicit an immunological response or allergic reaction; it also has excellent chemical resistance [24].

In this study, the tongue was pierced dorsoventrally, and this agrees with Peticolas et al. [22], who described two kinds of tongue piercing. The dorsoventral is the most common and harmless technique. The dorsolateral piercing in which, the implant is placed along the lateral edges of the tongue. Because the lateral tongue is highly vascularized and innervated, this is not a risk-free procedure.

In this study, the implant was placed in the midline of the tongue, anterior to the frenulum linguae and about 3–5 cm caudal to the tip of the tongue. This is the same surgical site as the previous tongue operation for prevention of self-sucking [7, 13]. However, tongue piercing too close to the tip would increase the risk of drifting of the rod towards the periphery of the tongue and ultimately rejection of the piercing. Furthermore, contraction of the tongue muscles causes the piercing to become more firmly embedded [25].

The piercing procedures in the present study were performed under sedation and local infiltration analgesia and this agrees with Farah and Harmon, [26] who said that piercing in people is not a painful procedure and carried out without anesthetic and mainly performed by nonmedical self-trained individuals or dental personnel with varying degrees of proficiency.

Safety was the most important factor in this study. However, only slight swelling around the piercing site of the tongue was recorded, and no long-term complications have been reported. In particular, no cases of the device’s dislodgement were recorded. And this is in contrast to Bentsen et al. [25], who reported the loss of the balls in medical tongue piercings and recommended using dental glue at the mounting of the balls in order to secure them further from loosening.

Because human oral microflora is diverse and abundant, it’s important to keep the pierced area clean, and antiseptic mouthwash must be used three to four times per day until the entire healing process is completed and if a patient complains of pain, oedema, and an inflammatory reaction from tongue piercing, the implant should be removed, local debridement performed, and antiseptic, anti-inflammatory, and antimicrobial treatment administered to hasten healing and resolve the problem [26].

Jornet et al. [27] performed tongue piercing in dogs and clinically found that none of the implants caused significant edema or hemorrhage, as well as none of the complications associated with human piercing. Pathologically, the piercing canal had been completely re-epithelialized at the expense of the healthy epithelium at the surgical wound’s edge.

In the present study only slight swelling around the piercing site of the tongue was recorded in all animals on the first 3 days after surgery and no other complications have also been reported. This may be attributed to the large number of salivary glands in ruminants that contribute to the production of large amounts of saliva, which can reach 100 L per day in adult cattle [10]. Saliva has been shown to hasten the wound healing process for a variety of reasons. Saliva produces moist environment, which improves the viability and activity of inflammatory cells, which are essential for wound healing; saliva has plenty of tissue factor, which helps blood clot faster. Furthermore, saliva includes a variety of peptides and proteins which provide protection against microbial pathogens by inhibiting bacterial adhesion and neutralizing microbial toxins [28, 29].

In the present study, we did not investigate the use of piercing technique to prevent inter- sucking. Inter-sucking was not recorded in our study because the housing system was tie-stall, which restricts the movement of the animal and each cow away from the other cattle, rather than a free-stall housing system, which allows cattle more opportunities to suck milk from the udders of other cattle. Loose housing allows the animals to engage in more abnormal behaviours, but space allowance, group size, and the general layout of the housing may limit some of the abnormal behaviours [3]. Therefore, a further study to investigate the use of piercing technique to prevent inter-sucking may be required.

## Conclusions

The piercing technique for prevention of self-sucking in cattle and buffaloes had a lot of advantages, like its quicker nature, as it can be considered a one-shot surgical technique, and its less invasive nature due to the preservation of tongue tissues with minimal intra-operative pain. Furthermore, the piercing technique was accepted by the owners due to its ability to solve the problem with low cost and rapid return of the operated animal to normal foot intake and productivity.

## Methods

### Ethical approval

All procedures in this study have been approved by the National Ethical Committee of the Faculty of Veterinary Medicine, Aswan University, Aswan, Egypt. All methods were carried out in accordance with relevant guidelines and regulations. All methods are reported in accordance with ARRIVE guidelines. Animals are cared for and used in research and education in accordance with Egyptian laws and OIE animal welfare guidelines. The owners signed an informed consent form to use the animals in the current study.

### Animals

The study was conducted on 26 cows and 4 buffaloes that belonged to private owners in Aswan governorate, Egypt, (ages ranged from 3 to 6 years) suffered from self-sucking behavioral disorder. These animals were selected for this technique after the failure of several trials of isolation, application of bull ring with spikes and after approval of the animals’ owners.

### Pre-operative clinical examinations

Before surgery, physiological parameters such as rectal temperature, heart rate, and respiratory rate were assessed to determine whether an individual animal was healthy enough to undergo this surgical technique.

### Anesthetic protocol

Tranquilization was acquired by administering xylazine HCl 2% (Xyla-Ject, Adwia Company, Egypt, injectable solution, xylazine hydrochloride 23.3 mg, eq. to 20 mg xylazine base) at a dose rate of 0.05 mg/kg body weight of the animal for operations that were performed in a recumbent position and at a dose rate of 0.01 mg/kg body weight of the animal for operations that were performed in a standing position. Lidocaine HCl 2% (Chemicals Company for El-Debeiky Pharma, Egypt) was locally infiltrated into the lingual submucosa of the operative site.

### Pre-operative measures

The oral cavity was flushed with povidone iodine solution 1% (Betadine-mouth wash, El- Nile Company for Pharmaceutical industries, Cairo, Egypt). The tongue was grasped gently, disinfected with povidone iodine solution 10%, and a tourniquet (made of rolled gauze) was circumferentially applied to the base of the tongue as close to the frenulum linguae as possible. The operative area on the dorsal surface of the tongue is marked with a pen, usually along the midline, anterior to the frenulum linguae and about 3–5 cm caudal to the tip of the tongue.

### The piercing instruments

**Fig. 1:**
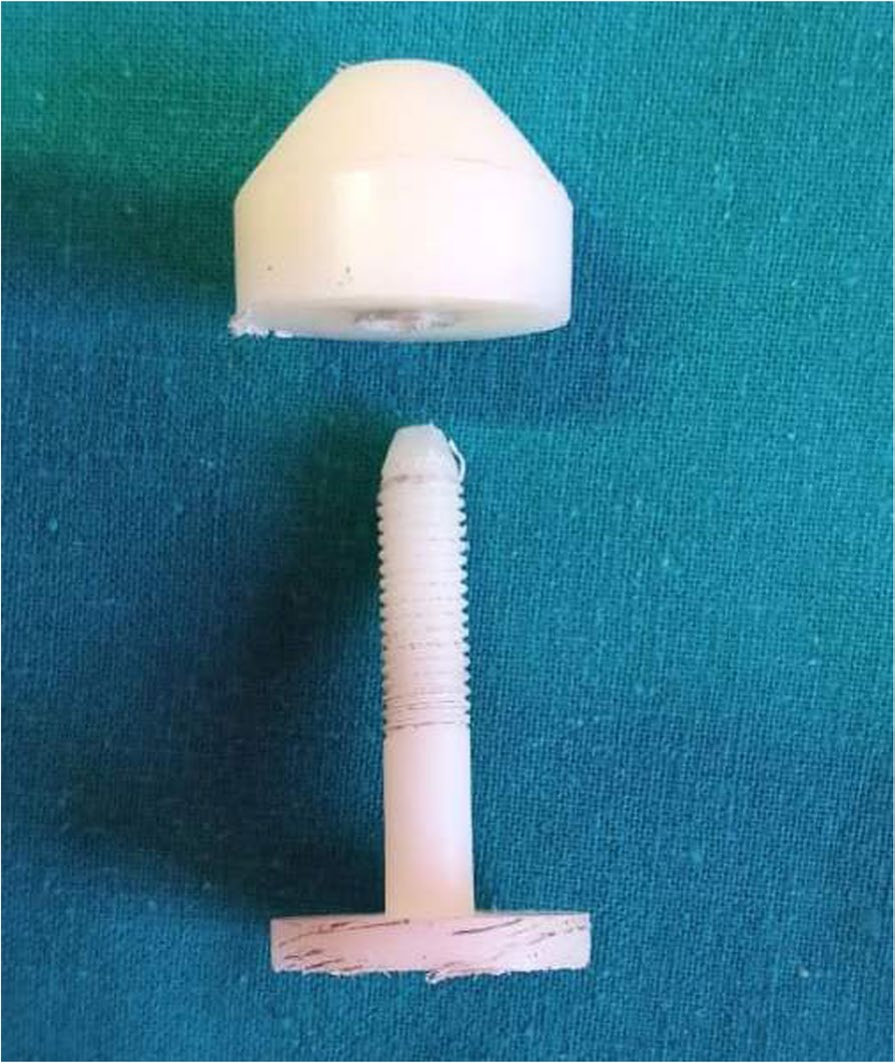
The implant consists of a barbell with two screws; one is fixed and the other is removable and made of polytetrafluoroethylene (PTFE), known as Teflon

Tongue piercings were performed with a sheep stainless trocar with cannula. The implant consists of a barbell with two screws; one is fixed and the other is removable. It was made of polytetrafluoroethylene (PTFE), known as Teflon, and was custom-made in the workshops of the Faculty of Engineering at Aswan University (Fig. 1).

### The piercing procedures

**Fig. 2:**
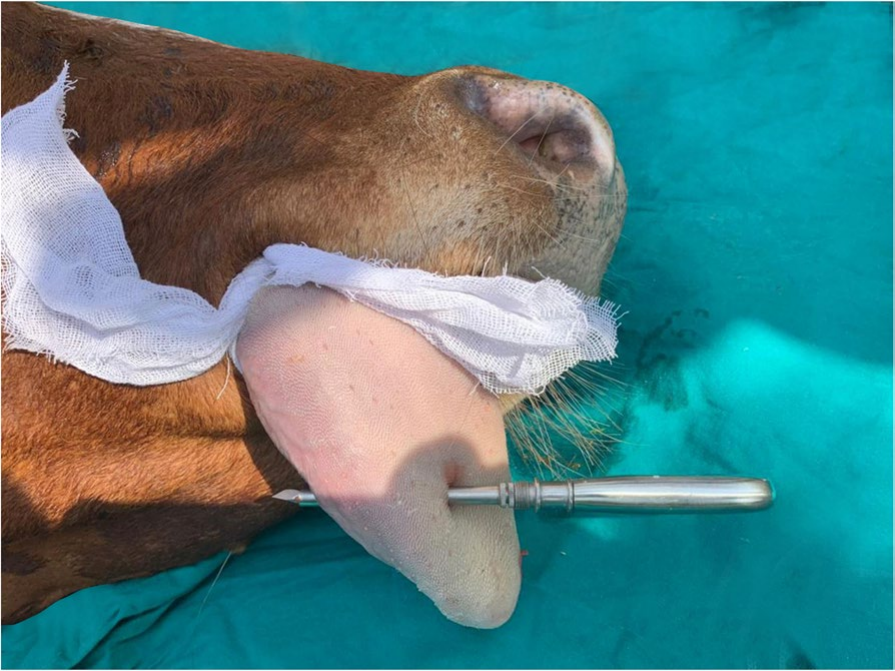
Trocarization of the tongue in the recumbent position

**Fig. 3:**
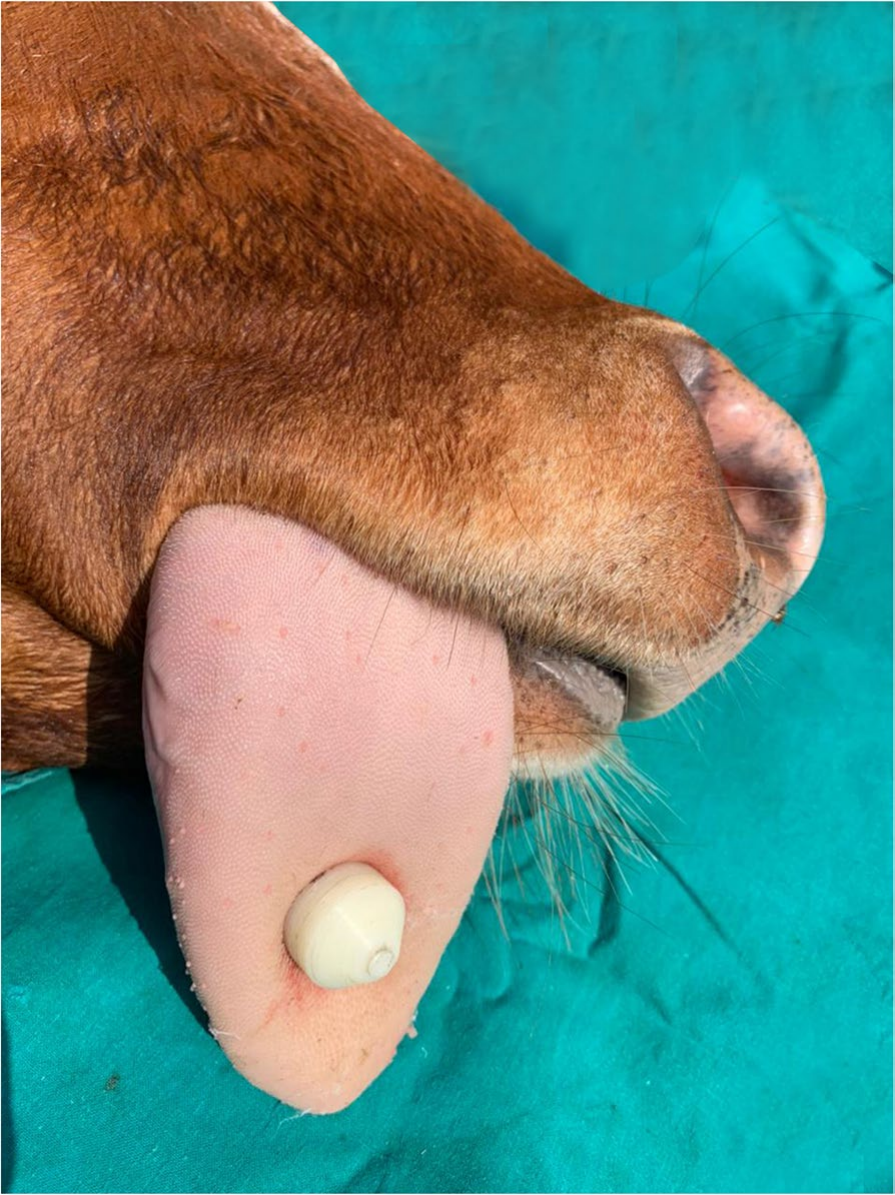
The implant from the dorsal surface of the tongue immediately after the operation

**Fig. 4:**
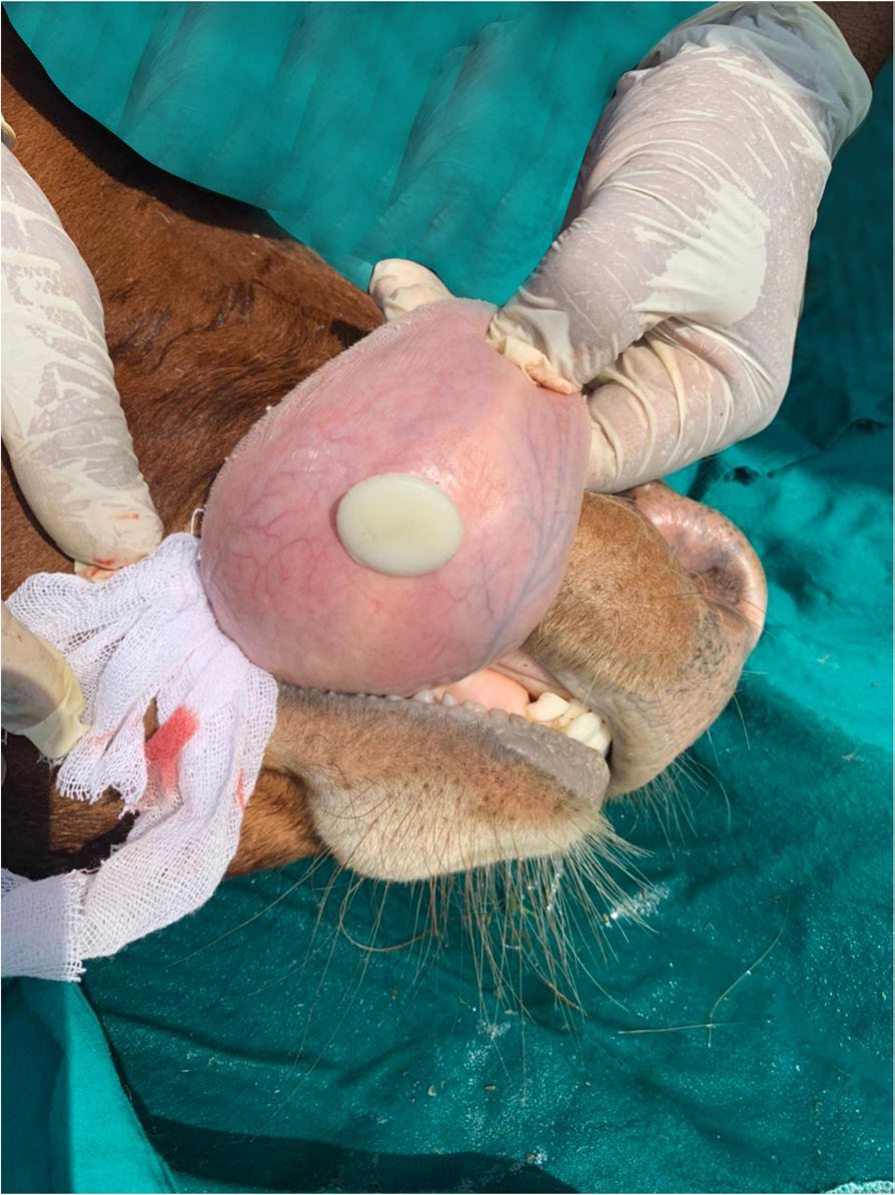
The implant from the ventral surface of the tongue immediately after the operation

The trocar and cannula pierced the tongue in a dorsal-ventral direction. The trocar was then removed and the barbell was inserted through the cannula traversing the tongue. Once the barbell was in place, the cannula was removed, and the removable screw was screwed into place with a pair of pliers and the bandage was removed (Figs. 2, 3 and 4).

### Post-operative measures

**Fig. 5:**
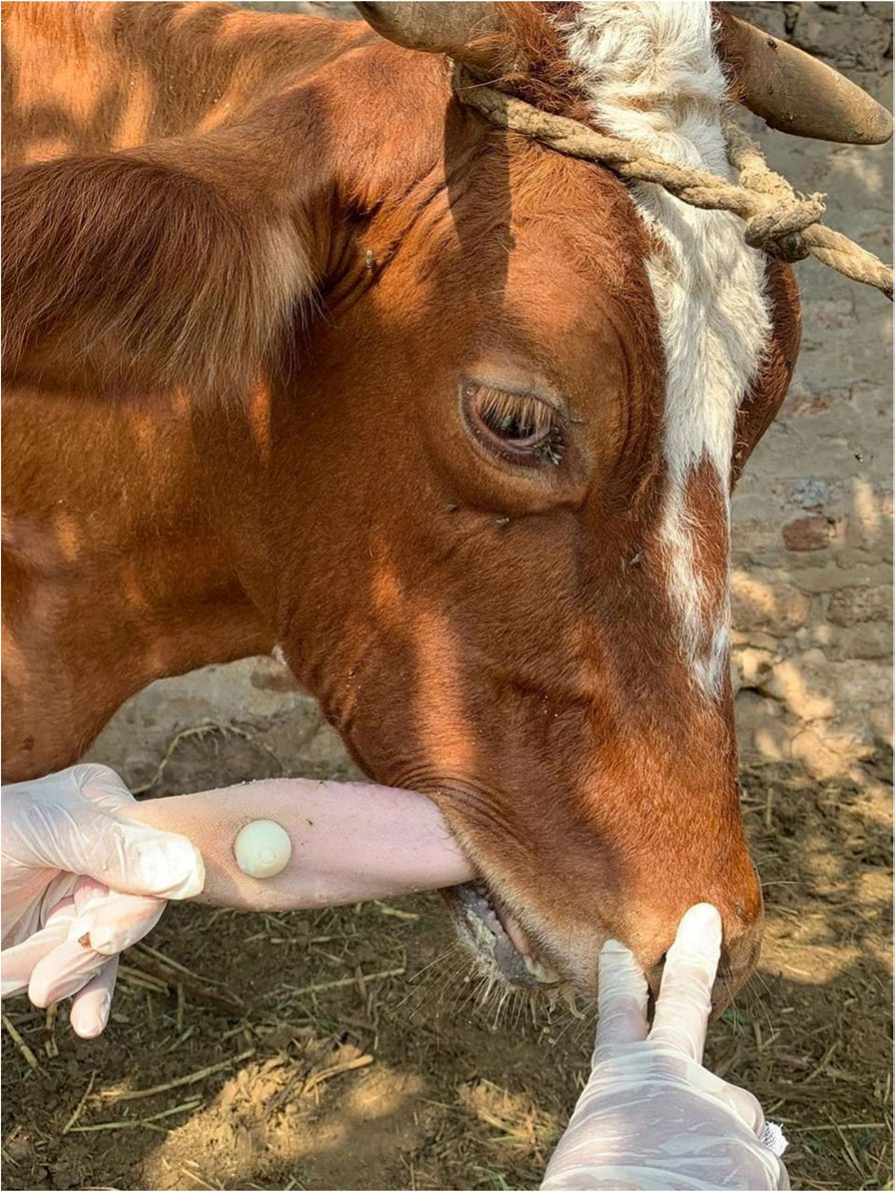
Tongue piercing after 6 months

The oral cavity was irrigated with povidone iodine solution 1% twice daily for three successive days. Animals were allowed to drink and eat freely after the operation. The owners were instructed to observe the treated animals and document any attempts by the treated animals to suck themselves. All treated cases were followed for up to 6 months to see if there were any complications (Fig. 5).

## Data Availability

All data generated or analysed during this study are included in this published article.
